# Uphyloplot2: visualizing phylogenetic trees from single-cell RNA-seq data

**DOI:** 10.1186/s12864-021-07739-3

**Published:** 2021-06-05

**Authors:** Stefan Kurtenbach, Anthony M. Cruz, Daniel A. Rodriguez, Michael A. Durante, J. William Harbour

**Affiliations:** Sylvester Comprehensive Cancer Center, Interdisciplinary Stem Cell Institute, Bascom Palmer Eye Institute, University of Miami Miller School of Medicine, Miami, FL USA

## Abstract

**Background:**

Recent advances in single cell sequencing technologies allow for greater resolution in assessing tumor clonality using chromosome copy number variations (CNVs). While single cell DNA sequencing technologies are ideal to identify tumor sub-clones, they remain expensive and in contrast to single cell RNA-seq (scRNA-seq) methods are more limited in the data they generate. However, CNV data can be inferred from scRNA-seq and bulk RNA-seq, for which several tools have been developed, including inferCNV, CaSpER, and HoneyBADGER. Inferences regarding tumor clonality from CNV data (and other sources) are frequently visualized using phylogenetic plots, which previously required time-consuming and error-prone, manual analysis.

**Results:**

Here, we present Uphyloplot2, a python script that generates phylogenetic plots directly from inferred RNA-seq data, or any Newick formatted dendrogram file. The tool is publicly available at https://github.com/harbourlab/UPhyloplot2/.

**Conclusions:**

Uphyloplot2 is an easy-to-use tool to generate phylogenetic plots to depict tumor clonality from scRNA-seq data and other sources.

## Background

**Fig. 1:**
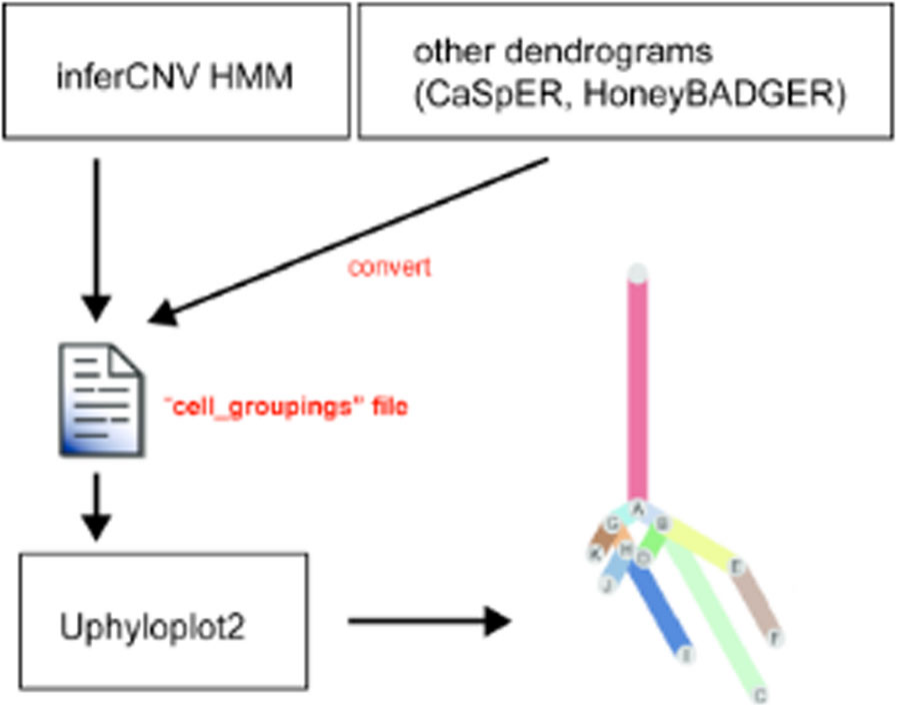
Workflow to generate phylogenetic trees with Uphyloplot2. “cell_groupings” files from inferCNV can be used directly. Alternatively, a conversion tool is included as part of the Uphyloplot2 package, which allows to convert any other Newick formatted dendrograms to a “cell_groupings” file.

Single cell RNA sequencing (scRNA-seq) has become an important new tool for studying gene expression in individual cells of heterogenous samples. While this technology is still maturing, it is already providing powerful new insights into normal and diseased tissue types [1, 2]. In particular, single cell technology has resulted in great strides in cancer research. A hallmark of cancer cells is aneuploidy and chromosomal copy number variations (CNVs), which often correlate with tumor aggressiveness [3–6]. CNVs can be used to identify subclones of tumor cells and to infer tumor evolution, which can have important clinical implications [7]. Single cell sequencing can be used to analyze subclonal tumor architecture at unprecedented resolution [1, 8]. While single cell DNA sequencing (scDNA-seq) is an emerging technique for this type of analysis, it is very expensive and yet to be optimized. Alternatively, CNVs can be inferred from scRNA-seq and bulk RNA-seq using applications such as inferCNV [9], HoneyBadger [10], and CaSpER [11]. Following, these applications cluster the inferred CNV patterns, allowing to define discrete subclones and infer tumor evolution. This approach for studying tumor clonality and evolution has been used successfully by our group and others [8, 12]. Tumor evolution is commonly visualized with phylogenetic plots, where the length of tree branches is proportional to the number of cells in each subclone. This, in contrast to plotting the dendrogram files, allows for a simple and intuitive representation of tumor evolution. Until now, such visualization required time-consuming and error-prone manual curation. Here we describe a new tool called Uphyloplot2. This program uses inferCNV output files to generate phylogenetic plots depicting tumor evolution, and also works with any other Newick formatted dendrogram files such as those derived from HoneyBADGER and CaSpER (Fig. 1).

### Implementation

Uphyloplot2 was written entirely in Python 3 to enable pipeline integration, customization, and platform independence.

### Availability and requirements

Project name: Uphyloplot2. Project home page: https://github.com/harbourlab/UPhyloplot2/. Operating system(s): Platform independent. Programming language: Python. Other requirements: None. License: GNU General Public License v3.0. Any restrictions to use by non-academics: No.

## Results

**Fig. 2:**
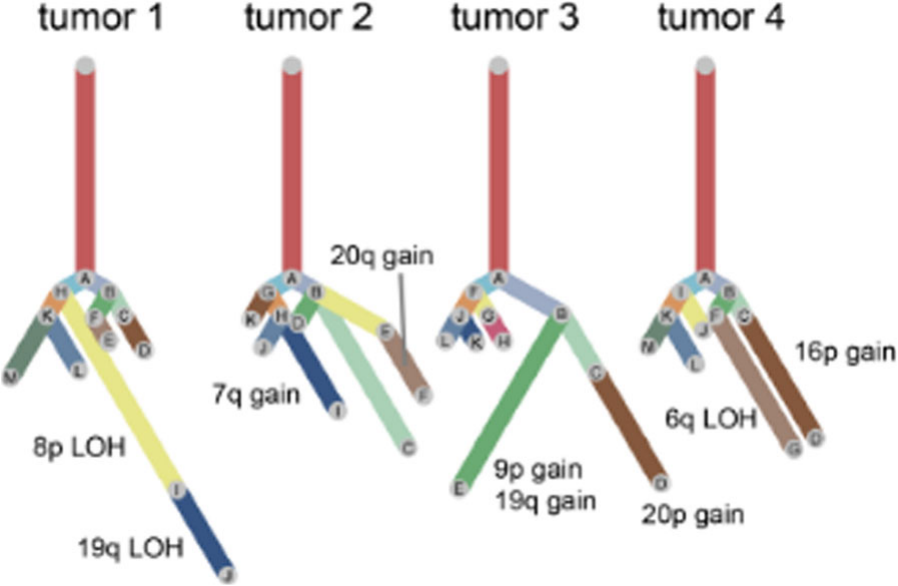
Example output of Uphyloplot2 using four input files. Branch lengths are proportional to the number of cells present in each subclone. Chromosomal gains and losses were inferred manually in addition.

To infer tumor clonality/evolution from scRNA-seq data, we first ran the inferCNV [9] pipeline on four uveal melanoma tumor samples [8] to infer CNVs from RNA-seq and cluster cells into subclones. inferCNV must be run with “HMM” to generate a “HMM_CNV_predictions.*.cell_groupings” file, which contains information on cell clusters. Following, reference cells (normal controls) were removed from that file manually before plotting. Uphyloplot2 can plot multiple trees at once and will plot all files placed in the “Input” directory in one figure. In the example above, we used all four “.cell_groupings” files to produce the four phylogenetic trees depicted in Fig. 2. The first branch (seen in red) always has the same length and is introduced to depict the evolution of normal cells to tumor cells. All following branches are labeled with letters corresponding to distinct tumor subclones. The branch length correlates with the number of cells in the respective subclone. For instance, in tumor 1 most cells are found in cluster “I” and “J”, where “J” is predicted to have directly evolved from “I”. Subsequently, more detailed information on which chromosomal regions were gained and lost for each subclone can be obtained from the “.HMM_CNV_predictions.*.pred_cnv_regions.dat” file. For example, cells in cluster “J” have lost part of chromosome 19q, in addition to the chromosome 8p loss found in cluster “I”. As can be seen in this simple example, sub-clonality of the four tumor samples differs substantially, and indicates the presence of multiple evolutionary branches.

Uphyloplot2 was designed to work directly with the “.cell_groupings” output from inferCNV after removing reference cells. Additionally, Uphyloplot2 can plot user derived, Newick formatted dendrogram files, for instance exported from HoneyBadger, CaSpER, or inferCNV if preferred. Using dendrogram files requires additional processing steps: In brief, using R the dendrogram has to be exported in a “Newick” format. Second, the Uphyloplot2 folder contains a python script called “newick_input.py”, which can be used to convert the Newick file to a “.cell_groupings” file. Once the “.cell_groupings” files are generated, they can be used as outlined above. A detailed user guide is available on the Uphyloplot2 GitHub page.

## Conclusions

The python script presented here allows to plot phylogenetic trees of tumor subclones from inferCNV output files and other Newick formatted dendrograms. The output files generated are true Scalable Vector Graphics (SVG) files, enabling easy attribute editing like colors, lengths, or angles in any SVG editor, while maintaining high resolution. Depending on the datasets, some branches might overlap in the figure, however, these can easily be rotated for visual clarity. In contrast to algorithms that estimate molecular time from whole-genome sequencing data using mutations [13], the use of CNVs to infer clonality and tumor evolution is more complex because some chromosomal segments are selectively altered while others occur through massive genome reorganization such as chromothripsis [14, 15], chromoplexy [16] and anaphase catastrophe [17]. It is important to note that Uphyloplot2 evolutionary plots might not represent molecular time accurately. Uphyloplot2 constructs trees with subclone branch lengths proportional to the number of cells in each subclone. New methodologies are also being developed for analyzing single cell CNV and single cell mutation data [18]. In summary, we present an automated tool for generating phylogenetic trees from scRNA-seq data that allows the visualization of tumor subclones and heterogeneity.

## Data Availability

The tool is publicly available at https://github.com/harbourlab/UPhyloplot2/, including example data.

## Abbreviations

CNVs: Copy number variations
scRNA-seq: Single cell RNA-seq
scDNA-seq: Single cell DNA sequencing
SVG: Scalable Vector Graphics
